# Translocator protein (18 kDa) regulates the microglial phenotype in Parkinson’s disease through P47

**DOI:** 10.1080/21655979.2022.2068754

**Published:** 2022-04-27

**Authors:** Xue Xue, Rui Duan, Guoyan Zheng, Hucheng Chen, Weiwei Zhang, Liang Shi

**Affiliations:** a Department of Nuclear Medicine, Nanjing First Hospital, Nanjing Medical University, Nanjing, Jiangsu, China; b Department of Neurology, Nanjing First Hospital, Nanjing Medical University, Nanjing, Jiangsu, China; c Department of Pathogenic Biology, Nanjing University of Chinese Medicine, Nanjing, Jiangsu, China

**Keywords:** Translocator protein, P47, oxidative stress, neuroinflammation, microglia phenotype, Parkinson’s disease

## Abstract

Numerous studies have suggested that the phenotypic transformation of microglia plays a role in the pathogenesis of Parkinson’s disease (PD). Translocator protein (TSPO) is an 18 kDa translocator membrane protein that acts as a marker of neuroinflammation and suppresses neuroinflammation; however, its underlying mechanism remains unclear. Although TSPO ligands were found to be protective in several neurodegenerative paradigms, few studies have evaluated their effects on microglial polarization, and underlying mechanisms need to be explored. In the present study, we examined the effects of TSPO and PK11195, a TSPO ligand, on lipopolysaccharide (LPS)+interferon (IFN)-γ-induced inflammatory factors and oxidative stress in microglia using enzyme-linked immunosorbent assay. The effect of TSPO and PK11195 on LPS+IFN-γ-induced microglial cell apoptosis was examined using immunofluorescence (IF), flow cytometry, and western blotting. The interaction between TSPO and P47 was investigated using IF and co-immunoprecipitation analysis. *In vivo* experiments confirmed the influence of TSPO and its ligand on motility, a-Syn, and dopaminergic neuronal damage. Our findings indicate that TSPO may regulate the microglial phenotype in PD via P47, suggesting a potential role in anti-PD therapy.

## Graphical abstract

**Figure:**
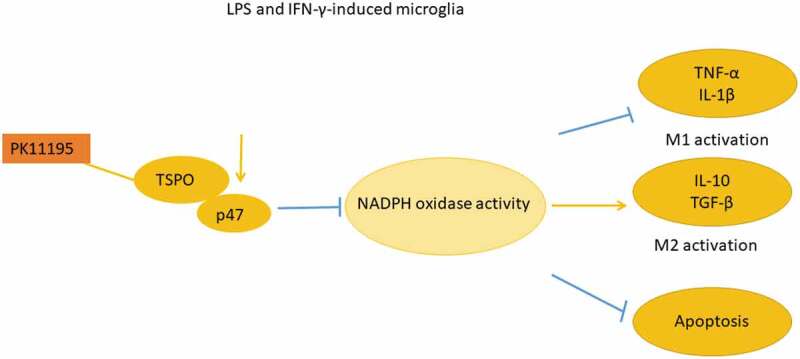

## Highlights

- TSPO knockdown promotes the M2 phenotype in BV2 cells.
- TSPO may modulate microglial activation in PD via P47.
- TSPO knockdown and PK11195 reduce dopaminergic neuronal damage.

## 1. Introduction

Parkinson’s disease (PD) is a common neurological disorder [1] that affects approximately 2–3% of the global population aged ˃65 years; however, its underlying etiology and pathogenesis remain poorly understood [2]. The neuropathological hallmarks of PD include degeneration of dopaminergic neurons, Lewy body deposition, and glial activation in the substantia nigra densa (SNc) [3]. Accumulating evidence suggests that microglial dysfunction plays a key role in the pathogenesis and progression of PD.

Microglia are innate immune macrophages specific to the central nervous system (CNS) [4] that perform various functions critical for the development, homeostasis, and repair of the CNS. In their static state, microglia constantly assess the brain microenvironment and detect changes that might interfere with normal brain functioning through phagocytosis and immune monitoring [5]. Nevertheless, microglia with disturbed immune homeostasis may phenotypically develop two polarized states: pro-inflammatory and anti-inflammatory (M1 and M2 phenotypes, respectively) [6]. Notably, both microglial phenotypes play prominent roles in inflammatory diseases. The M1 phenotype generates inducible nitric oxide synthase (iNOS) and secretes pro-inflammatory cytokines, triggering an immune response [7]. Conversely, the anti-inflammatory M2 phenotype typically promotes inflammation resolution and tissue repair, thereby ameliorating tissue damage. The M2 phenotype was shown to express arginase 1, facilitate tissue repair, and secrete anti-inflammatory cytokines [8]. However, the mechanism through which glia maintain their physiological state remains poorly understood. Nonetheless, it is likely that reducing microglia-mediated hyperinflammation and inducing the predominant anti-inflammatory microglial phenotype would afford effective therapies for controlling neuroinflammation and potentially treating neurodegenerative diseases.

Overproduction of reactive oxygen species (ROS) induces oxidative stress, which is known to mediate the pathology and pathogenesis of PD [9]. In the brain, endogenous ROS are primarily derived from nicotinamide adenine dinucleotide phosphate (NADPH) oxidase (NOX) [10], an enzyme responsible for superoxide production [11] and comprising cytoplasmic subunits p47^phox^, p67^phox^, p40^phox^, small GTPase Rac, gp91^phox,^ and p22^phox^ [12]. Upon activation, phosphorylated p47 reportedly initiates cytoplasmic complexes and promotes the assembly of activated NOX2 complexes [13]. P47^phox^ acts as an organizer of NOX2-activated phagocytic oxidase subunits. PD has been receiving growing attention [14]; however, the molecular mechanisms underlying microglial polarization in PD remain unclear.

Transporter protein (TSPO), an 18 kDa protein on the outer mitochondrial membrane, is widely employed as a preclinical and clinical biomarker of injury and neuroinflammation in the brain and can reportedly detect different brain lesions [15]. It should be noted that while TSPO expression is nearly undetectable in normal brain neurons, it markedly and selectively increases at primary and secondary sites of brain injury and neuroinflammation [16]. The expression of TSPO was found to significantly increase in microglia and astrocytes during neuroinflammatory and neurodegenerative diseases such as PD. In addition, TSPO is a marker of microglial activation [17]. Recent studies have shown that TSPO participates in the regulation of glial cell oxidative metabolism and inflammatory responses, and its effect is correlated with NADPH oxidase [18]. High TSPO expression can be associated with ROS resistance, whereas ROS production was shown to increase following TSPO knockdown [19]. These findings indicated that TSPO plays a vital role in the antioxidant response pathway. Another study has reported that the administration of NADPH oxidase inhibitors can modulate TSPO-induced ROS production. Although confocal detection of TPSO co-localization with gp91^phox^ has been reported, it remains unclear whether TSPO directly interacts with NOX2 intracellular subunits and its downstream antioxidant signaling pathway [20]. Recent findings indicate that TSPO ligands can be used to attenuate neuroinflammation and neurotoxicity in rodent models [21]. However, no previous study has reported the TPSO-mediated modulation of microglial phenotypes and its effect on PD. Therefore, in the present study, we aimed to determine the role of TSPO and its ligands on the microglial phenotype. Furthermore, we explored the relevance of this effect on the underlying pathological mechanism of PD, aiming to probe the possible molecular mechanisms involved. These findings will further clarify our current understanding of PD and provide new targets to develop neuroprotective agents that suppress TSPO.

## 2. Methods

### 2.1 Cell culture

Murine microglial BV2 cells were cultured with Dulbecco’s Modified Eagle Medium (Gibco/Thermo Fisher Scientific, MA, USA) supplemented with fetal bovine serum (10%, Gibco) and penicillin-streptomycin (1%, Thermo Fisher Scientific) in a humidified, 5% CO_2_ incubator at 37°C.

BV-2 cells were exposed to 100 ng/mL lipopolysaccharide (LPS; Sigma-Aldrich) and interferon (IFN)-γ (20 ng/mL; Peprotech) for 24 h. Subsequently, BV2 cells were transfected with TSPO siRNA and siRNA-NC (100 nM; Genechem) for 6 h using Lipofectamine 2000 (Invitrogen) according to the manufacturer’s instructions and harvested 12 h after transfection. Cells were divided into two groups: LPS+IFN-γ+ si-NC and LPS+IFN-γ+ si-TSPO groups. The cells were then treated with PK11195 (0.5 Μm; Sigma-Aldrich) for 6 h (LPS+IFN-γ+ PK11195 and LPS+IFN-γ+ si-TSPO+PK11195 groups, respectively).

### 2.2 Enzyme-linked immunosorbent assay (ELISA)

The levels of interleukin (IL)-1β, IL-6, IL-10, transforming growth (TGF)-β, and tumor necrosis factor (TNF)-α in the cell culture supernatant of each group were detected using ELISA (Boster). Perform as requested by the manufacturer. The optical density of each well was measured at 450 nm using a microtiter plate reader (Thermo Fisher). NADPH activity in the supernatant of each group was detected using an NADPH oxidase kit (Nanjing Jiancheng Bioengineering Institute).

### 2.3 Immunofluorescence (IF)

Briefly, BV2 microglial cells were treated with paraformaldehyde (4%, 15 min), followed by treatment with bovine serum albumin (BSA; 5%) and Triton X-100 (0.1%) for 30 min. The samples were then treated with IBA1 (1:1000), TSPO (1:1000), and P47 (1:1000) antibodies at 37°C for 2 h. Subsequently, the samples were treated with secondary antibodies Alexa Fluor 594, 488, and 674 (Thermo Fisher) for 45 min (room temperature). Finally, the samples were treated with DAPI at room temperature for 15 min and imaged using a confocal microscope (Olympus, Tokyo, Japan) [22].

### 2.4 TUNEL assay

Apoptosis was detected using a one-step TUNEL assay kit (Beyotime), according to the manufacturer’s instructions. Nuclei were stained with DAPI, and fluorescence images were captured using a fluorescence microscope. The number of fluorescence-positive cells and total cells in each group were counted, and the percentage of apoptotic cells in each group was calculated as the number of positive cells to total cells.

### 2.5 Flow cytometry

Briefly, BV-2 cells were inoculated in the culture dish and treated according to the experimental group design after cells reached 70–80% confluency. After washing twice with cold phosphate-buffered saline (PBS), the cells were centrifuged at 1000 rpm for 5 min to obtain a single-cell suspension (1 × 10^6^/mL). Subsequently, a 2-fold volume of pre-cooled (−20°C) 95% ethanol solution (Fuyu Chemical) was gradually added for fixation. Next, 100 U/mL ribonuclease A (RNase A; BD Biosciences) was added and mixed by shaking in a constant temperature water bath shaker for 30 min (37°C), followed by treatment with propidium iodide (PI; 10 μg/mL) staining solution containing Triton X-100, protected from light at 4°C for 30 min. The percentage of apoptotic cells was analyzed using the CellQuest software based on the appearance of subdiploid peaks (apoptotic peaks) on the DNA histogram [23].

### 2.6 Immunoprecipitation assay

After transfection, BV-2 cells underwent lysis, and the supernatant was collected as the total cell protein lysate (input sample). Then, immunoprecipitation was performed with antibodies and protein A/G beads for 4 h at 4°C. Western blot analysis was performed as described in subsection 2.10.

### 2.7 Animals and methyl-4-phenyl-1,2,3,6-tetrahydropyridine (MPTP) treatment

Male C57BL/6 J mice (5–6 weeks old, weighing 18–22 g) were purchased from Nanjing Medical University Animal Core (Nanjing, China) and housed at 22–25°C and 40–70% humidity with free access to food and water under a 12-h light/dark cycle. All animal procedures were performed in accordance with the guidelines of the National Institutes of Health’s Guide for the Care and Use of Laboratory Animals.

To construct the PD model, mice were administered intraperitoneal (i.p.) MPTP injection (30 mg/kg; 5 days); the control group was administered normal saline (30 mg/kg). One week after successful modeling, open field and pole tests were performed to assess behavior.

### 2.8 Animal behavioral assessment

In the open field test, mice were monitored using an automated flex field/open field activity system (ANY-maze) in a 50 × 50 × 30 cm white opaque plastic box. Mice were placed in the central square of the box and recorded for 5 min. Experimental data were automatically transmitted to a computer for further analysis.

In the pole test, mice were placed on a vertical wooden post (50 cm high, 1 cm diameter, rough surface) with their head facing upward. Mice were housed in the instrument room for two days prior to the test for environmental acclimatization and were tested on the third day. The total time (T-total, up to the point where the mice reached the ground with all four paws) and turn time (T-turn, where mice turned completely headdownward) were recorded. The experimenter performed behavioral tests blinded to the animal group, and all tests were performed in triplicate.

### 2.9 Immunohistochemistry (IHC)

Briefly, brain sections were rinsed with PBS, washed with H_2_O_2_ (3%,10 min), and subsequently incubated with Triton X-100 (0.3%) and BSA (5%) supplemented with PBS for 1 h. Then, tissues were treated with tyrosine hydroxylase (TH) antibody (1:1,000; Sigma) or alpha-synuclein (α-Syn) antibody (1:500; BD) in PBS (5% BSA) overnight at 4°C, followed by incubation with secondary antibody at 25°C (2 h), and finally with diaminobenzidine (DAB; 5 min) for staining. Images were observed under an Olympus microscope, and photographs were captured. The number of TH- and α-Syn-positive cells was calculated using the MicroBrightField Stereo-Investigator software (MicroBrightField) [24].

### 2.10 Western blotting

Briefly, brain tissue and microglia were homogenized by sonication and incubated in RIPA lysis buffer (Thermo Fisher). The samples were isolated by 12% sodium dodecyl sulfate-polyacrylamide gel electrophoresis (SDS-PAGE), then transferred to PVDF membranes (Millipore). Subsequently, the bands were blocked and then incubated overnight at 4°C with primary antibodies against Bcl-2 (1:1,000, Bioworld), caspase-3 (1:500; Abcam), P47 (1:1,000; Abcam), TSPO (1:2,000; Thermo Fisher), and GAPDH (1:2,000; Abcam). Next, membranes were treated with horseradish peroxidase (HRP)-conjugated secondary antibody. The bands were then assayed with chemiluminescence (ECL; Millipore). After cleaning, the membranes were scanned and analyzed with the Image Quant LAS 4000 chemiluminescent imaging system (GE Healthcare) and ECL Western blot detection reagent Pierce™ ECL (Thermo). Quantitative analysis was performed using ImageJ software (National Institutes of Health, Bethesda, MD).

### 2.11 Statistical analysis

Data values are presented as mean ± standard error of the mean (SEM). Data were subjected to one-way ANOVA using SPSS 20.0 (IBM Corp., Armonk, NY, USA), followed by Tukey’s post-hoc test. Values were considered significant at P < 0.05.

## 3. Results

The aim of this study was to investigate the phenotypic changes of microglia in PD by TSPO. Firstly we examined the inflammatory factors (IL-1β, IL-6, IL-10; TNF-α, TGF-β) and NADPH oxidase activity secreted by microglia M1/M2 phenotype activation. Next, we performed apoptosis-related analysis and confirmed that TSPO and its ligand PK11195 play a vital role in microglia phenotype regulation. To explore possible underlying mechanisms, CO-IP and co-localization were used to reveal TSPO-P47 interactions. Finally, we constructed in vivo models to validate the neuroprotective effects of TSPO and its ligand in mice.

### 3.1 Effect of TSPO on LPS- and IFN-γ-induced interleukin levels in BV2 cells

**Figure 1.**
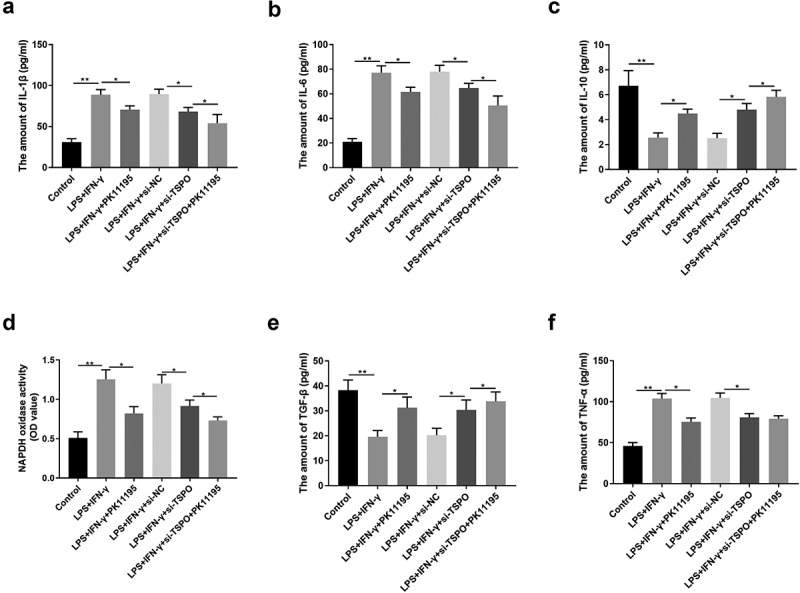
TSPO and PK11195 regulated the levels of IL-1β, IL-6, IL-10, NADPH, TGF-β, and TNF-α of LPS and IFN-γ-induced microglia. (a) The expression of IL-1β in BV2 cells was detected by ELISA. (b) The expression of IL-6 in BV2 cells was detected by ELISA. (c) The expression of IL-10 in BV2 cells was detected by ELISA. (d) The NADPH activity in BV2 cells was detected by ELISA. (e) The expression of TGF-β in BV2 cells was detected by ELISA. (f) The expression of TNF-α in BV2 cells was detected by ELISA. (;P < 0.05, ;;P < 0.01).

To determine the effect of TSPO and its ligand PK11195 on inflammatory responses in microglia, we measured interleukin levels using ELISA. The results revealed that LPS+IFN‐γ treatment substantially increased IL‐1β and IL-6 expression when compared with the control group; however, these expression levels were significantly decreased following PK11195 treatment (Fig. 1A-B). Likewise, TPSO knockdown following LPS+IFN-γ stimulation effectively reduced IL-1β and IL-6 expression, with PK11195 treatment affording superior effects. As shown in Fig 1C, altered IL-10 expression exhibited an opposite trend to that of IL-1β and IL-6. We observed that IL-10 expression was lower in the LPS+IFN-γ group and relatively higher in the LPS+IFN-γ+ PK11195 group than that in the LPS+IFN-γ+ si-TSPO group.

### 3.2 Effect of TSPO on LPS- and IFN-γ-induced TNF-α, TGF-β, and NADPH levels in BV2 cells

We further examined the effect of TSPO and its ligand PK11195 on ROS production in microglia, and hence determined interleukin levels using ELISA. NADPH activity (Fig. 1D) was increased following LPS- and IFN-γ administration; PK11195 reversed the elevated NADPH levels in LPS+IFN-γ-induced cells. In addition, si-TSPOv reduced NADPH levels, and PK11195 coactivated with si-TSPO further diminished NADPH oxidase activity.

Following LPS and IFN-γ administration, TGF-β was downregulated, while TNF-α was upregulated; treatment with PK11195 treatment could reverse these changes. si-TSPO demonstrated a similar effect to PK11195, demonstrating a superior effect on TGF-β on combination treatment; however, no significant difference in TNF-α expression was observed (Fig. 1E-F).

### 3.3 TPSO knockdown inhibits LPS- and IFN-γ-induced apoptosis in BV2 cells

**Figure 2.**
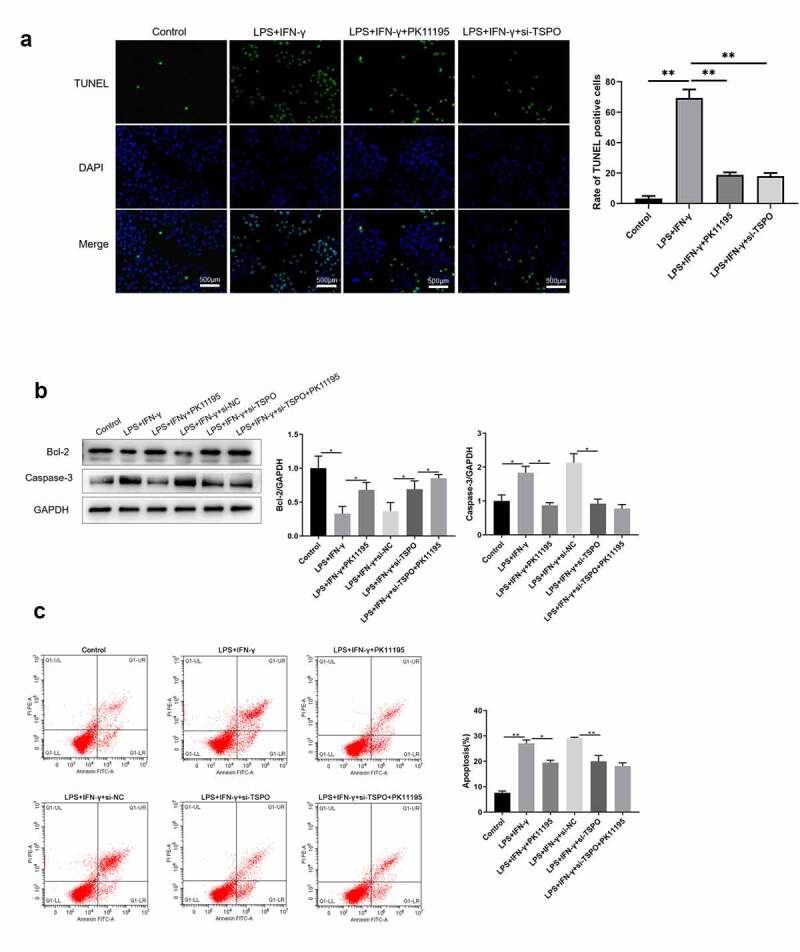
Knockdown of TSPO and PK11195 treatment partly protects microglia from LPS and IFN-γ induced apoptosis. (a) Cell apoptosis is analyzed using TUNEL staining. (b) Bcl-2 and caspase3 protein expression are measured using western blotting. (c) Flow cytometry analysis of apoptotic BV-2 cells. (;P < 0.05, ;;P < 0.01).

We next examined whether TSPO and its ligand PK11195 could affect LPS and IFN-γ-induced apoptosis by performing a TUNEL assay. As shown in Fig. 2A, LPS and IFN-γ stimulation dramatically enhanced apoptotic cells when compared with the control group, determined by TUNEL-positive staining. Conversely, the number of apoptosis-positive cells was markedly reduced in both the LPS+IFN-γ+ PK11195 and LPS+IFN-γ+ si-TSPO groups. Next, apoptosis-related proteins were detected. Western blot analysis (Fig. 2B) revealed that LPS and IFN-γ stimulation significantly upregulated caspase3 protein expression in microglia. However, Bcl-2 protein expression was significantly downregulated in LPS- and IFN-γ-stimulated cells. Intriguingly, LPS and IFN-γ induced apoptosis, as well as altered apoptosis-related protein expression, were alleviated following treatment with PK11195 or si-TSPO transfection. Flow cytometric analysis revealed that BV2 cells transfected with TSPO siRNA were less susceptible to LPS- and IFN-γ-induced apoptosis, exhibiting a lower apoptosis rate than siRNA-NC transfected control cells (Fig. 2C).

### 3.4 TSPO interacts with P47 in BV2 cells

**Figure 3.**
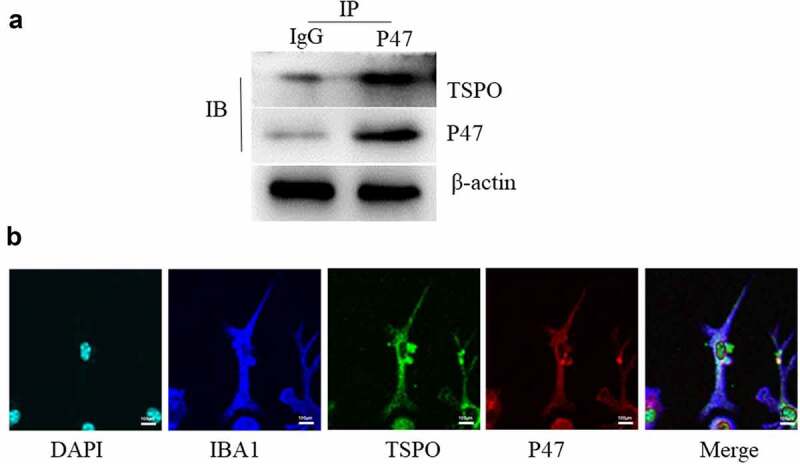
TSPO binds P47. (a) BV2 cells were lysed and immunoprecipitation was carried out with indicated antibodies. The immunocomplexes were subjected to western blot analysis. (b) Immunofluorescence imaging of IBA1, TSPO, P47 and DAPI was captured on the confocal microscope.

To further elucidate the mechanism of TSPO regulation, Co-IP experiments were performed to determine whether TSPO and P47 interact with each other. Endogenous TSPO was co-immunoprecipitated with endogenous P47 in BV2 cells (Fig. 3A). Confocal analysis of IBA1-TSPO-P47 staining confirmed the presence of TSPO and P47 co-labeling in glial cells (Fig. 3B).

### 3.5 PK11195 restores motor dysfunction and rescue the dopaminergic neuronal damage of MPTP/p PD model mice

**Figure 4.**
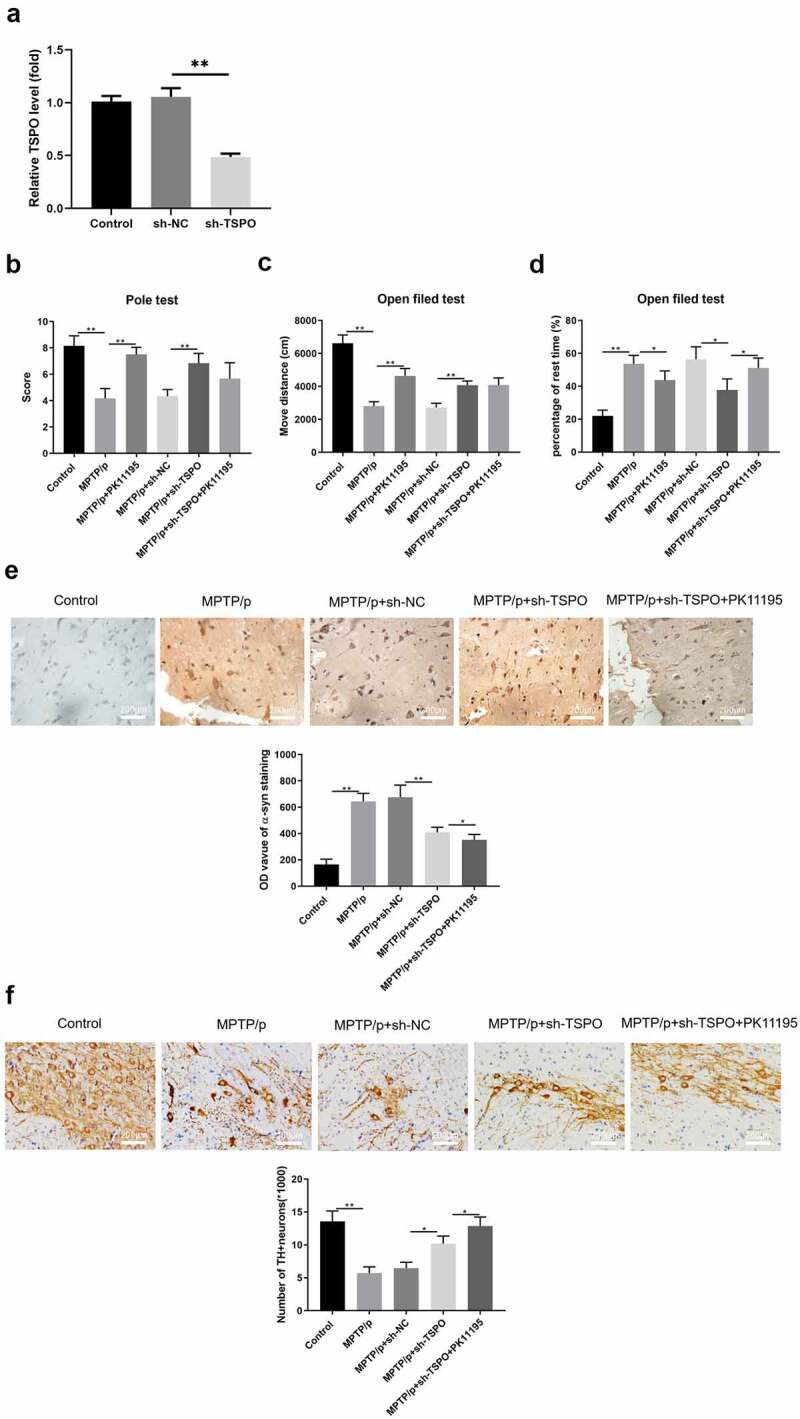
Knockdown of TSPO and PK11195 treatment ameliorated motor dysfunction and dopaminergic neuronal function of MPTP/p PD model mice. (a) The score of turn around (time of turning) and descend a pole (time of climbing) were recorded for the pole test. (b) The move distance was recorded for the open field test. (c) The rest time was recorded for the open field test. (d) Immunohistochemistry of α-Syn staining. (e) Immunohistochemistry of dopaminergic staining.(;P < 0.05, ;;P < 0.01).

To delineate the potential *in vivo* contribution of TSPO, we generated an MPTP/p PD mouse model. The knockdown efficiency of TSPO is demonstrated in Fig. 4A, and the results indicated that sh-TSPO could effectively reduce TSPO expression. Based on the observed results, we found that MPTP/p modeling decreased the pole test score (Fig. 4B), as well as reduced the distance traveled (Fig. 4C) and prolonged the rest period (Fig. 4D) in the open field; however, both PK11195 and si-TSPO injection potently reversed these changes. Interestingly, the combination of PK11195 and si-TSPO exhibited a superior effect in terms of improving rest time compared with respective administrations. Subsequently, we measured the pathological effects of TSPO on neuronal survival and α-syn aggregation in PD. Immunochemical staining revealed a significant increase in α-Syn expression in the MPTP/p-injured mice (Fig. 4E). Treatment with PK11195 and si-TSPO injections markedly decreased α-syn aggregation, which was further reduced on combination treatment with PK11195 with si-TSPO. Downregulated TH activity was also observed in mice challenged with MPTP/p, whereas PK11195 and si-TSPO upregulated TH activity (Fig. 4F).

## 4. Discussion

TSPO is primarily distributed in the neurons and microglia in the CNS. Upon activation of glial cells during nerve injury, TSPO is upregulated and could be a potential molecular marker of glial cell activation, representing a sensitive and specific quantitative indicator of neuropathological changes [25]. In recent years, the development of TSPO-targeted drugs has become a global research hotspot, promising the discovery of new areas in neuropharmacology and facilitating early diagnosis and intervention of neurodegenerative diseases [26]. PK11195 is the first positron emission tomography radioligand for the clinical detection of TSPO; however, its low brain tissue and highly nonspecific uptake, high lipid solubility, and elevated plasma protein binding rate are shortcomings known to impact its clinical application [27]. In addition, the application of TSPO ligands has been consistently reported to modulate NADPH oxidase activity and inhibit neuroinflammation, the precise mechanism of which remains unclear [28,29]. Another study has revealed that the use of the TSPO ligands PK11195 and Ro5-4864 can dramatically reduce neural damage and glial cell activity in Alzheimer’s disease transgenic mouse models [30]. Exogenous administration of TSPO ligands can inhibit inflammation and exert protective effects in animal models of brain injury and neuroinflammation [31]. The classically activated M1 type secretes pro-inflammatory factors (TNF-α and IL-1β) and ROS via the classical MAPK-NF-κB pathway, thereby causing neuronal death [32,33]. The selectively activated M2 type expresses pro-inflammatory factors such as IL-1, IL-10, and TGF-β by increasing AMP-activated protein kinase phosphorylation and peroxisome proliferator-activated receptor-gamma and Nrf2 intranuclear receptor transcription factors [34]. Therefore, the present study is the first to examine the effect of TSPO on the M1/M2 phenotypic transformation of microglia (Fig. 1A-C). The results suggest that TSPO knockdown, as well as administration of the PK11195 ligand, could differentially suppress the M2 phenotype in BV2 cells.

Recent studies have indicated that TSPO overexpression is linked to NADPH oxidase [20,35]. Some studies have implicated NADPH oxidase as a key factor in the phenotypic transition of microglia [36]. Inhibition or knockdown of NOX2 in brain injury models can downregulate the microglial MAPK-NF-κB signaling pathway, which in turn exhibits an M2 phenotype [37]. In Alzheimer’s disease models, inhibition of NADPH oxidase or knockdown of p47^phox^ was shown to induce microglial conversion from M1- to M2-type [38]. In addition, administration of NADPH oxidase inhibitors can modulate TSPO-induced ROS production, although confocal investigations revealed that TSPO colocalizes with gp91phox [29]. Specifically, NADPH oxidase expression is reportedly elevated in PD, and NADPH oxidase is considered both a major source of microglia-derived extracellular ROS and a critical mechanism for microglia-mediated neurotoxicity [39]. Thus, we next examined NADPH oxidase-related expression (Fig. 1D-F) and found that the TSPO ligand inhibited NADPH oxidase activity in the PD model. In addition, apoptosis-related assays were performed, revealing the protective effect of TSPO knockdown and its ligand in BV2 cells (Fig. 2). NADPH oxidase has been implicated in microglial phenotype regulation; however, it remains unclear whether it regulates M1/M2 conversion by altering the p47^phox^ conformation. Accordingly, we explored the possible underlying mechanisms., and as shown in Fig. 3, TSPO was found to interact with P47, thereby confirming that TSPO may modulate microglial activation in PD via P47.

In the PD brain, a considerable amount of M1-type microglia was found to accumulate in the proximity of dopaminergic neurons [40]. Damaged dopaminergic neurons release α-syn, matrix metalloproteinase 3 (MMP3), and neuromelanin in PD, which directly or indirectly trigger microglial activation. α-Syn was shown to be a prominent component of Lewy vesicles in the brains of patients with PD, and numerous studies have reported that α-Syn released extracellularly from dead dopaminergic neurons directly stimulates M1 microglia and NADPH oxidase activation, thereby inducing substantial ROS production and inflammatory factor expression [41]. During the pathological process of PD, M1/M2 microglia are mixed and dynamic, with the proportion of M1 microglia gradually increasing as the disease progresses, along with abundant α-syn accumulation [42]. Inhibition of certain inflammatory factors and pathways alone fails to comprehensively alleviate PD progression [7]. Therefore, reducing α-Syn accumulation may be a key switch that causes phenotypic changes in microglia and, in turn, regulates microglial M1 to M2 transition. Therefore, we validated the neuroprotective effects of TSPO and its ligands in mice using an *in vivo* PD model. Our findings implied that TSPO knockdown and PK11195 could improve the motility of MPTP acute PD model mice and reduce dopaminergic neuronal damage (Fig. 4).

## 5. Conclusions

In conclusion, TSPO knockdown and its ligand PK11195 could attenuate neuronal dopaminergic damage in an MPTP acute PD model, attenuate NADPH oxidase activity, and increase M2 inflammatory factors via P47. These findings not only broaden our understanding of progressive neuronal degeneration in PD but also provide new avenues for developing neuroprotective agents targeting TSPO inhibition.

## Supplementary Material

Supplemental Material
