# Supplementary figures and images for: Translocator protein (18 kDa) regulates the microglial phenotype in Parkinson’s disease through P47
Source: Bioengineered. 2022 Apr 27;13(4):11061–71. doi: 10.1080/21655979.2022.2068754 (PMC9208449; doi:10.1080/21655979.2022.2068754)

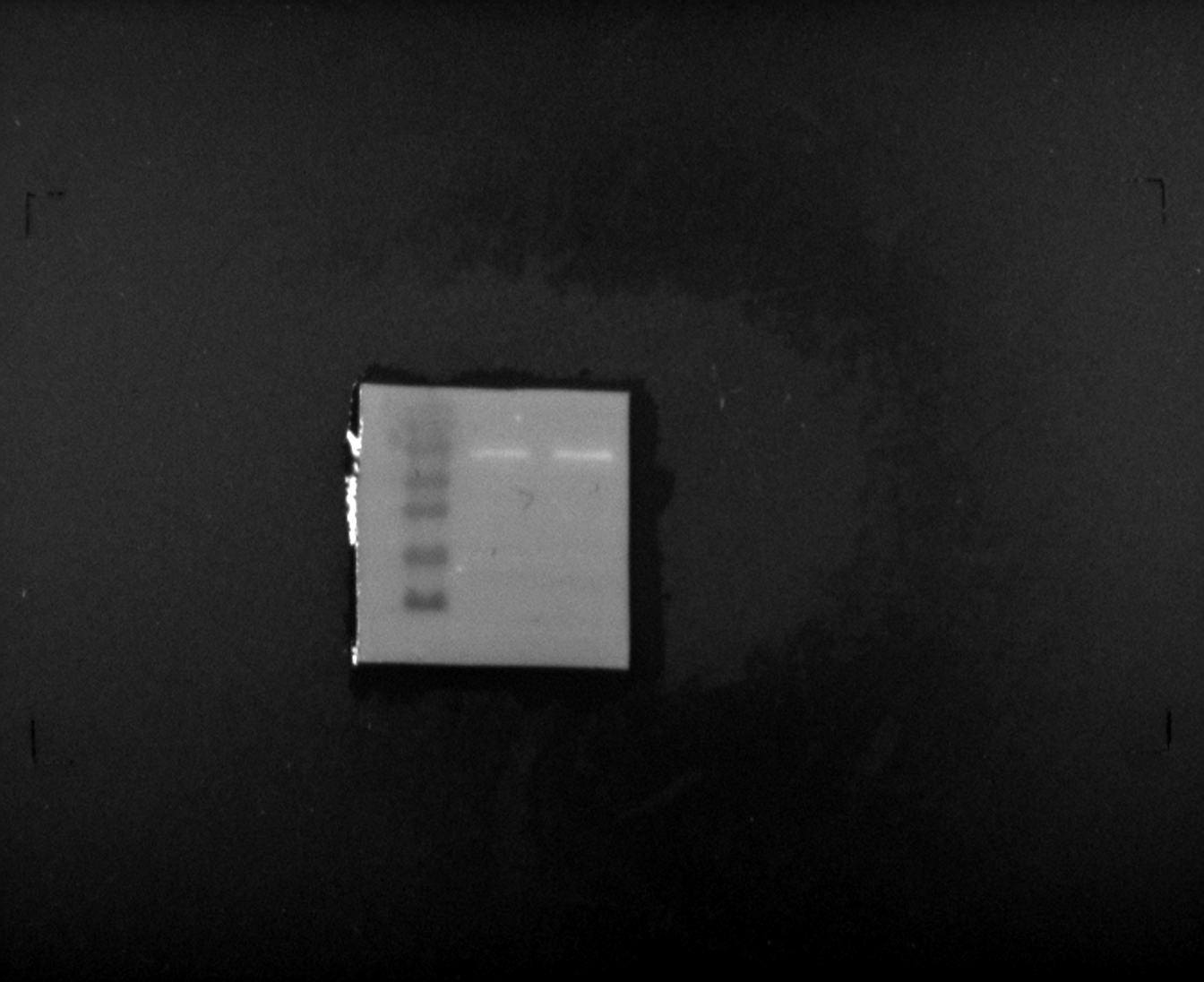

Supplement: Supplemental Material [file KBIE_A_2068754_SM7231.zip › supplementary/CO-IP/ACTIN (1).tif]

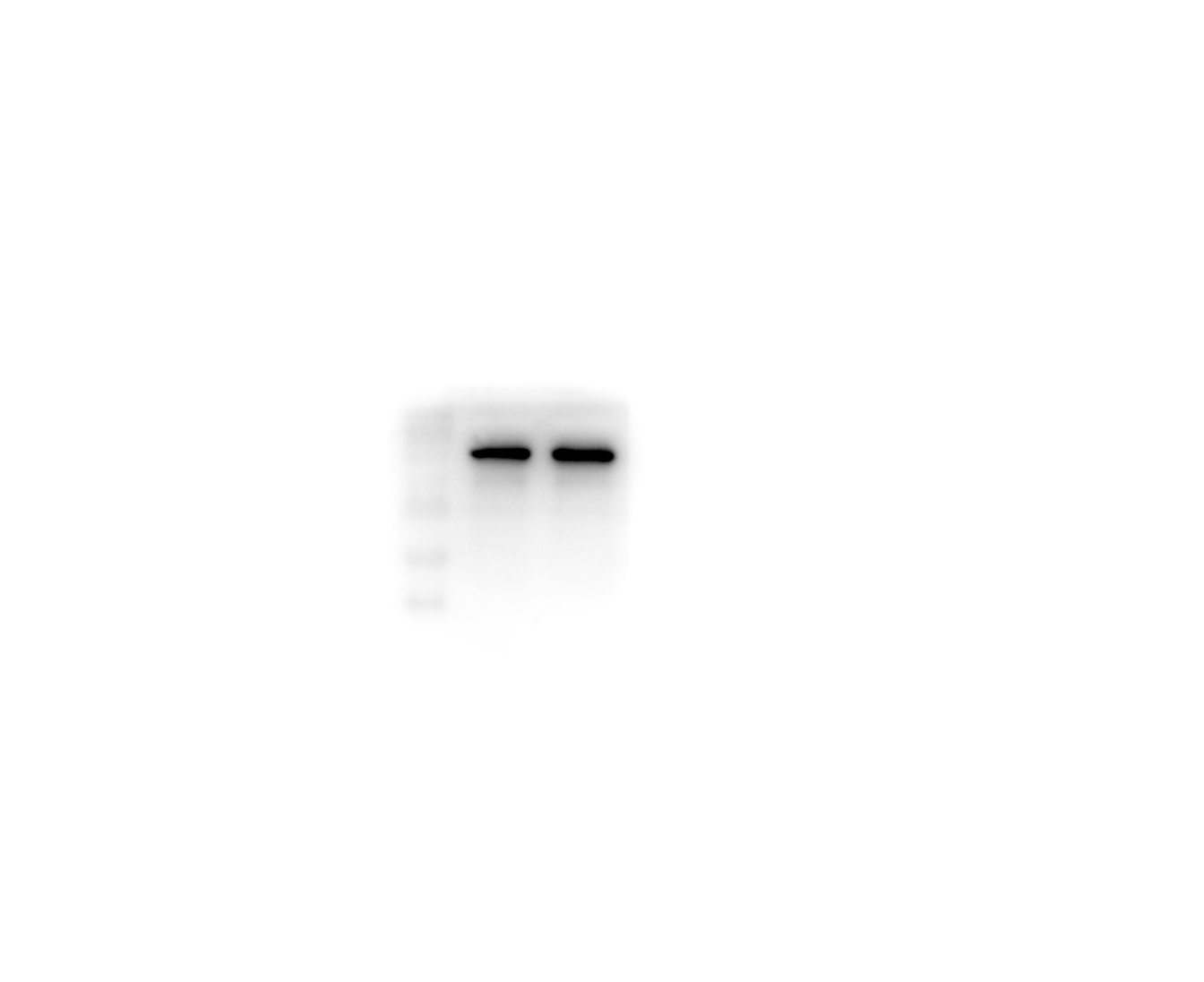

Supplement: Supplemental Material [file KBIE_A_2068754_SM7231.zip › supplementary/CO-IP/ACTIN (2).tif]

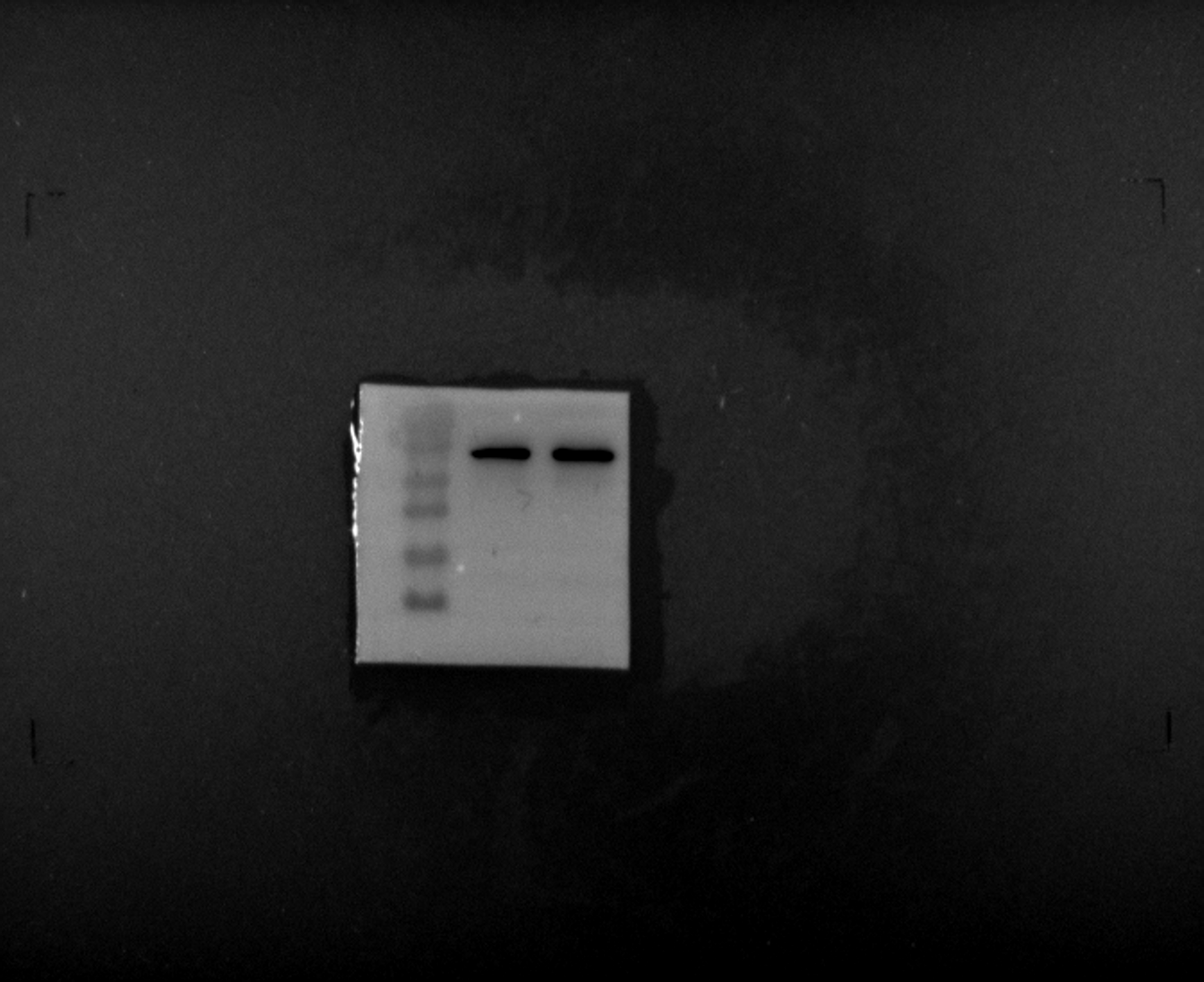

Supplement: Supplemental Material [file KBIE_A_2068754_SM7231.zip › supplementary/CO-IP/ACTIN (3).tif]

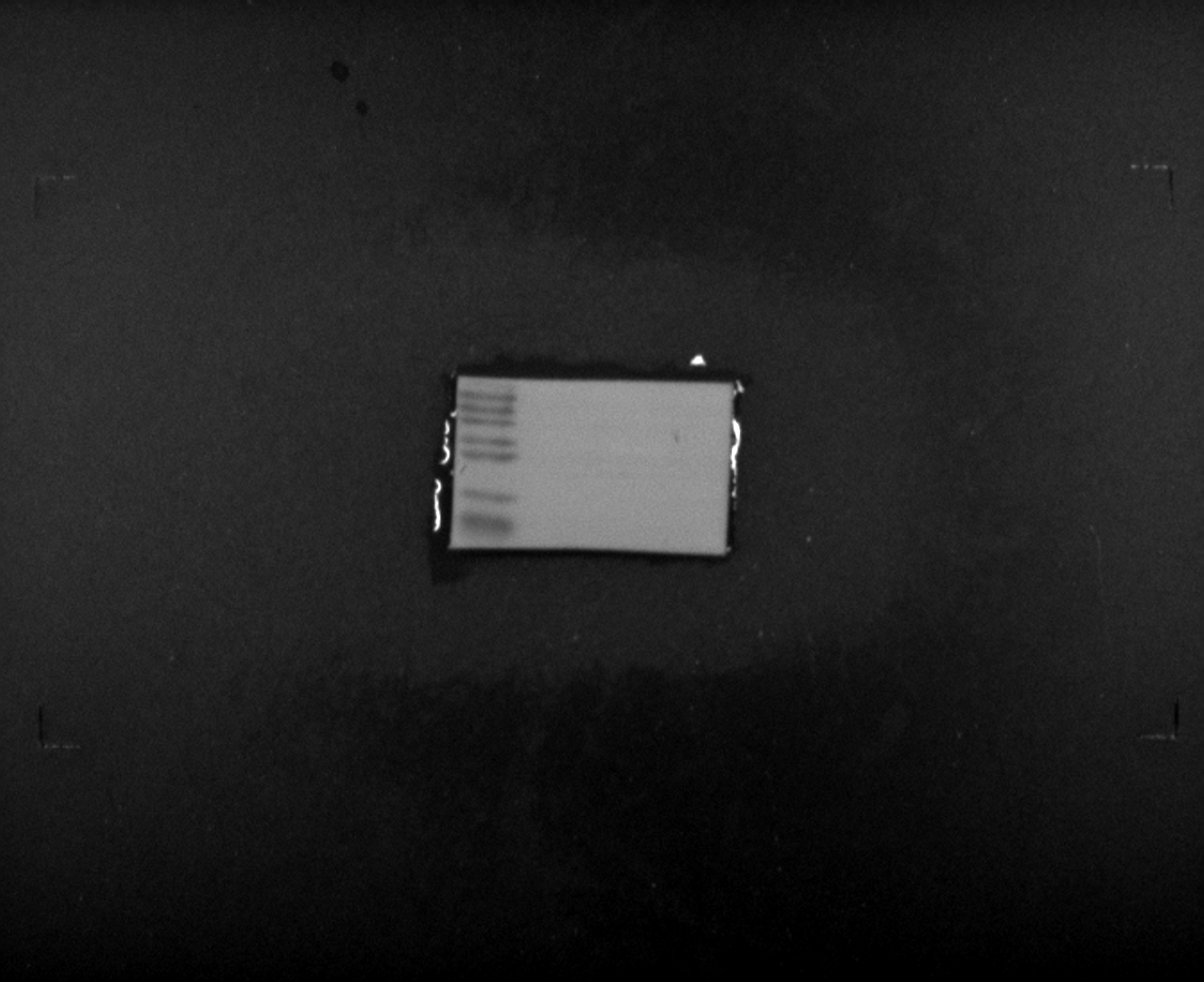

Supplement: Supplemental Material [file KBIE_A_2068754_SM7231.zip › supplementary/CO-IP/P47 (1).tif]

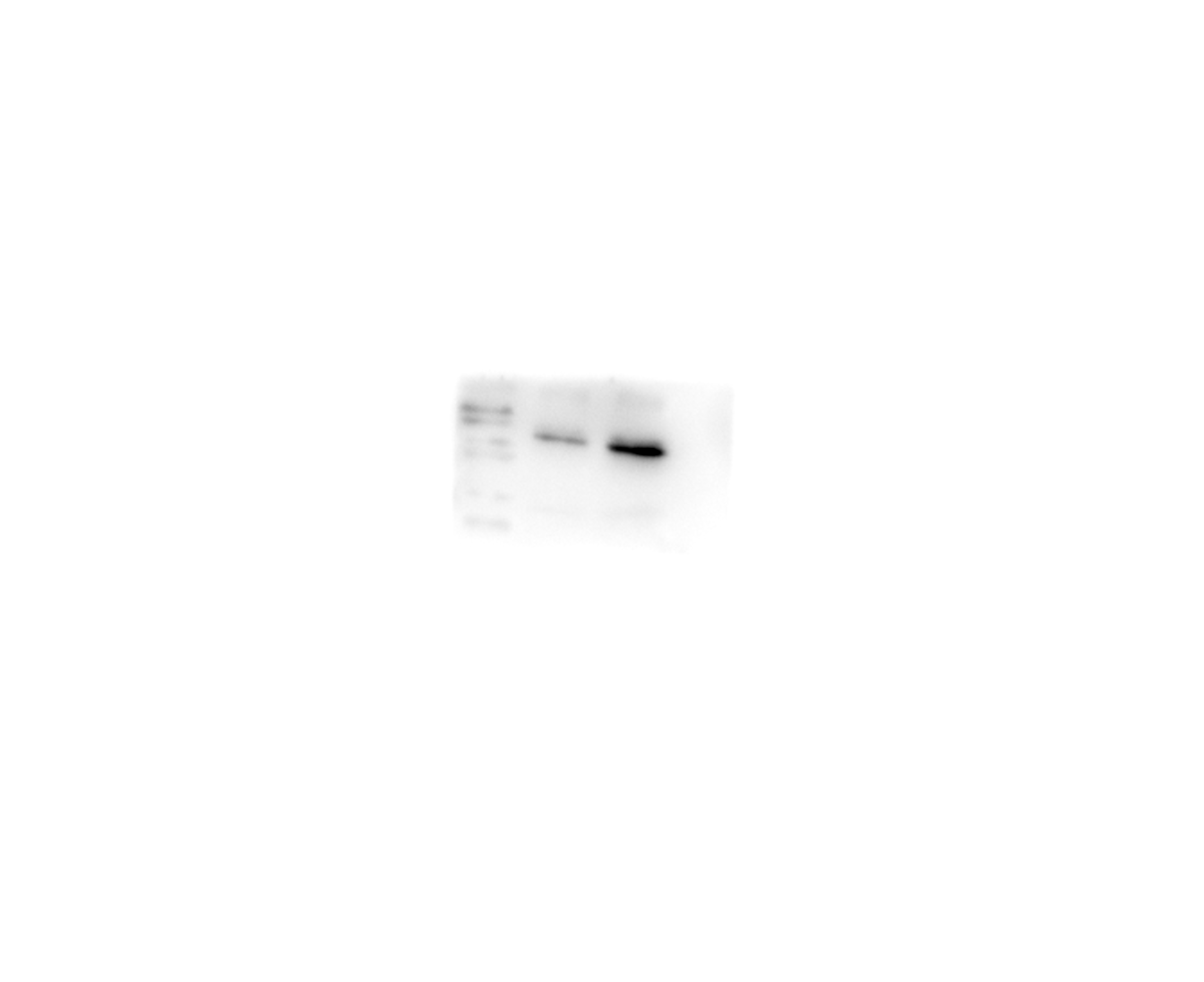

Supplement: Supplemental Material [file KBIE_A_2068754_SM7231.zip › supplementary/CO-IP/P47 (2).tif]

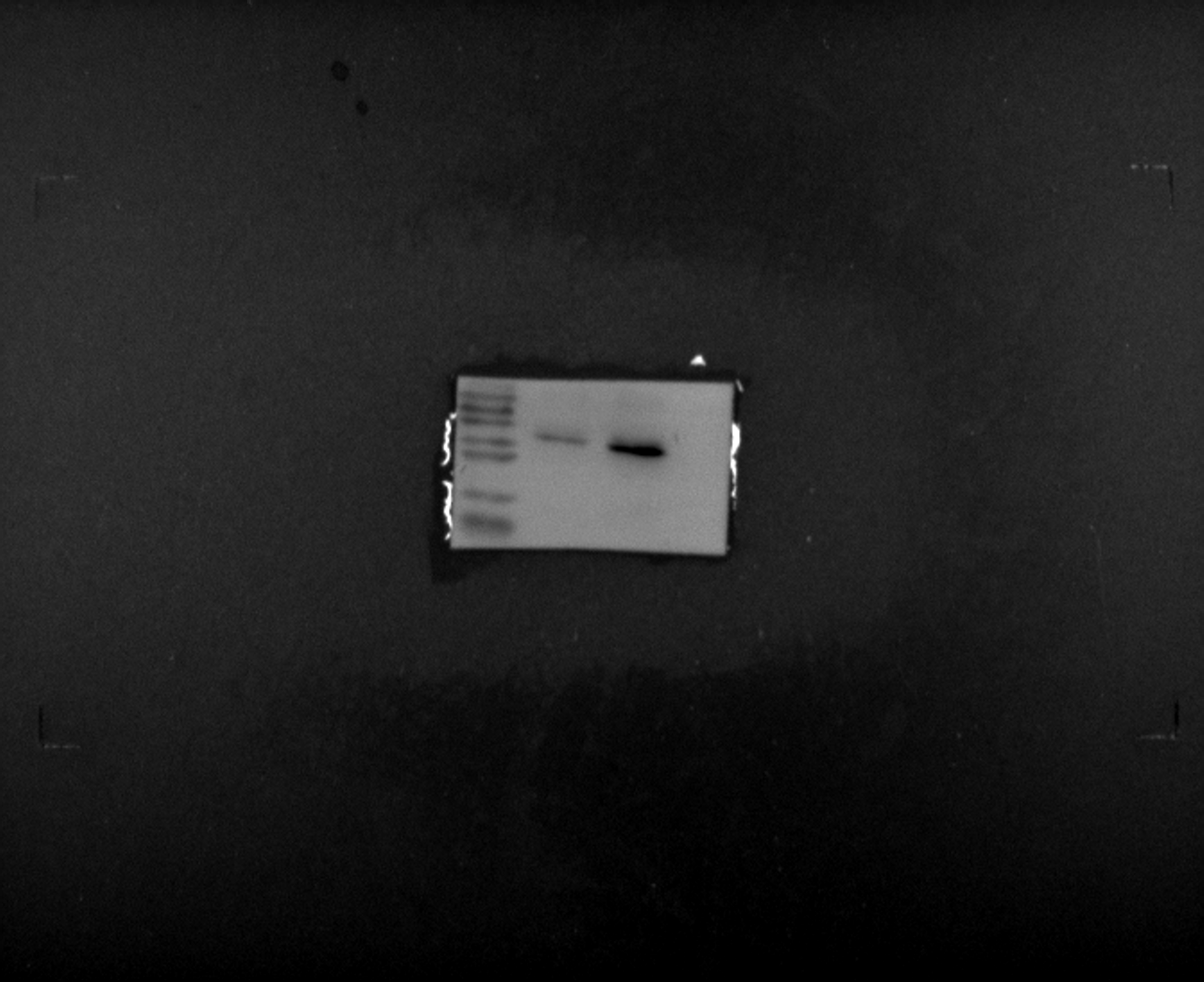

Supplement: Supplemental Material [file KBIE_A_2068754_SM7231.zip › supplementary/CO-IP/P47 (3).tif]

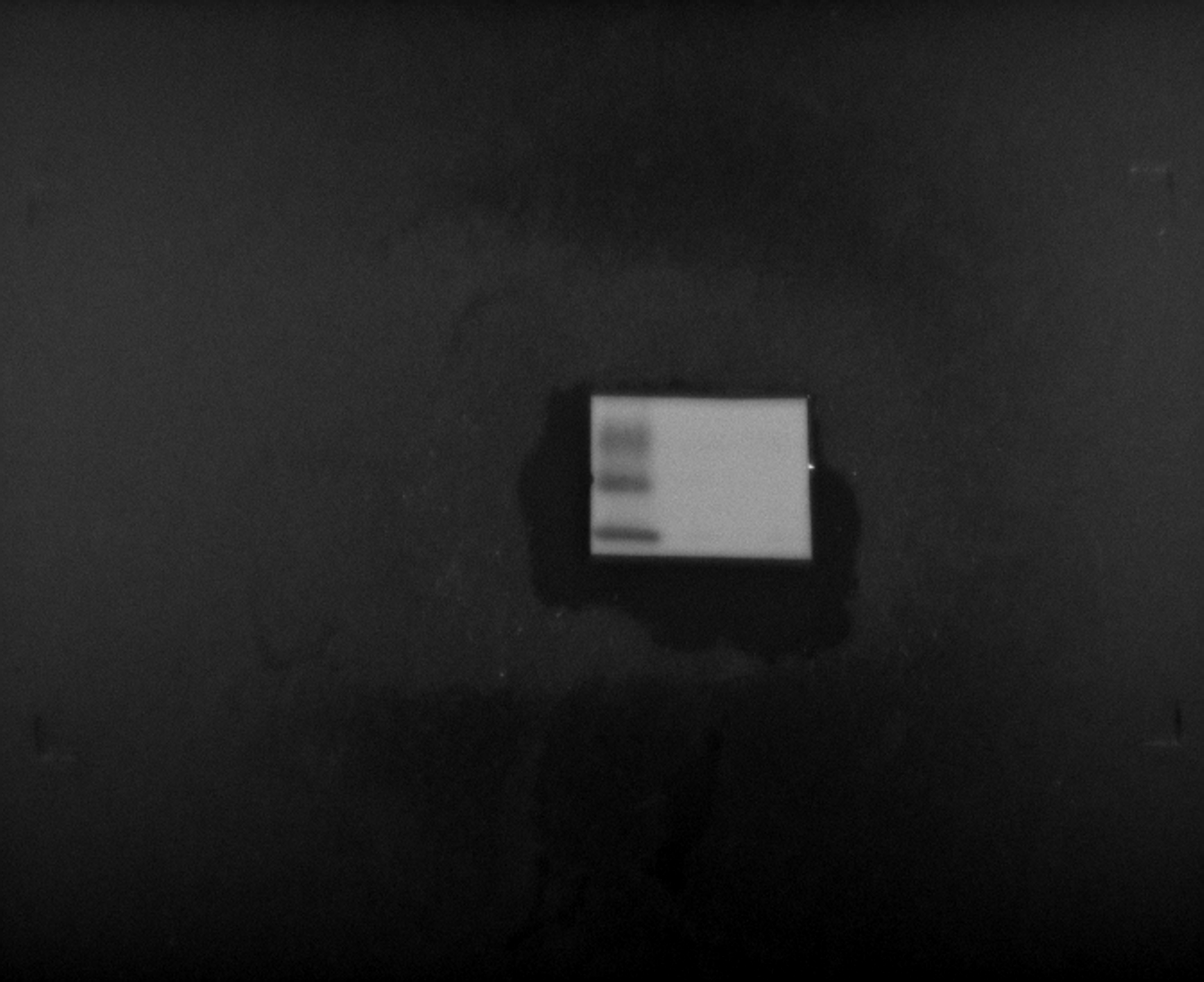

Supplement: Supplemental Material [file KBIE_A_2068754_SM7231.zip › supplementary/CO-IP/TSPO (1).tif]

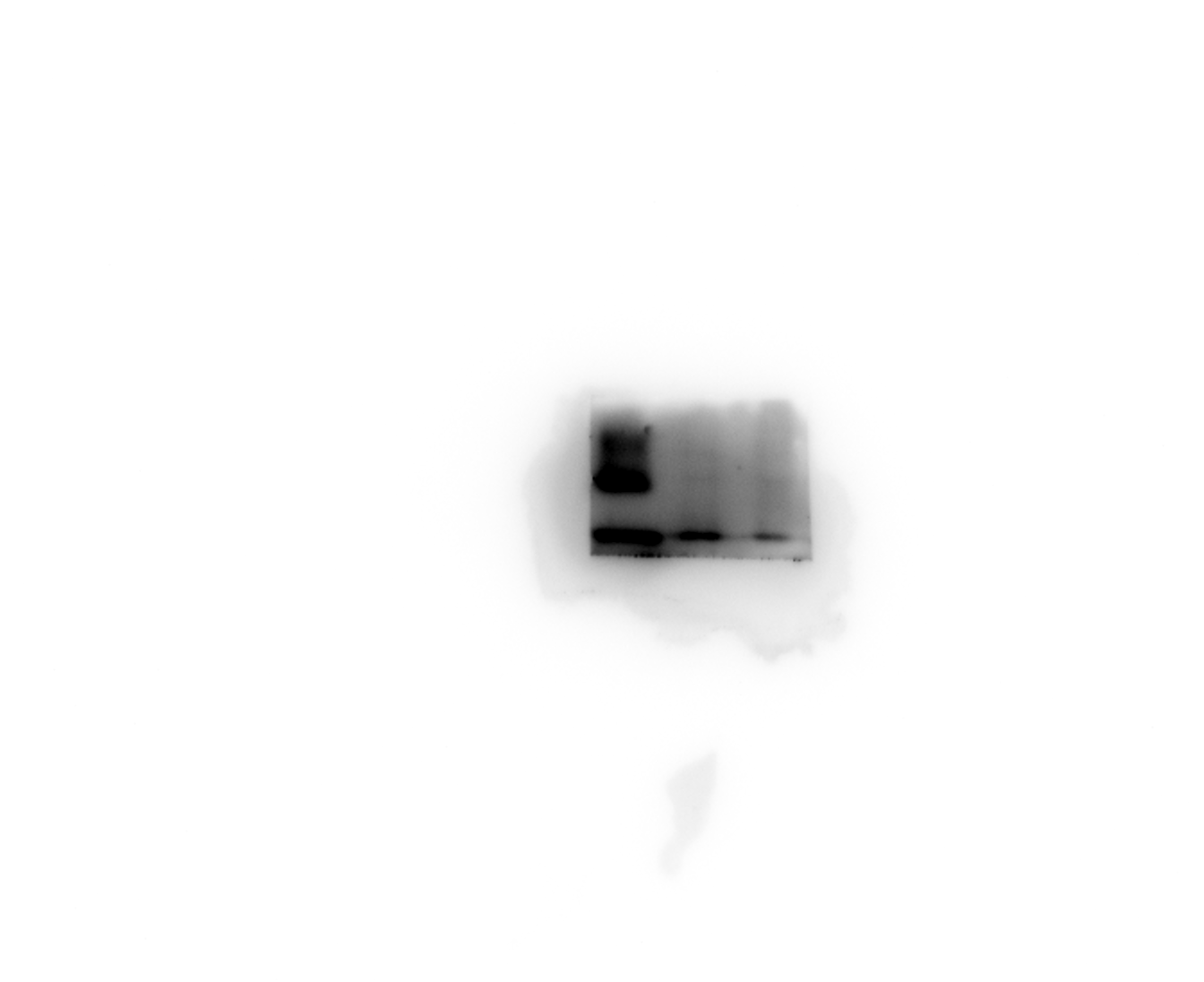

Supplement: Supplemental Material [file KBIE_A_2068754_SM7231.zip › supplementary/CO-IP/TSPO (2).tif]

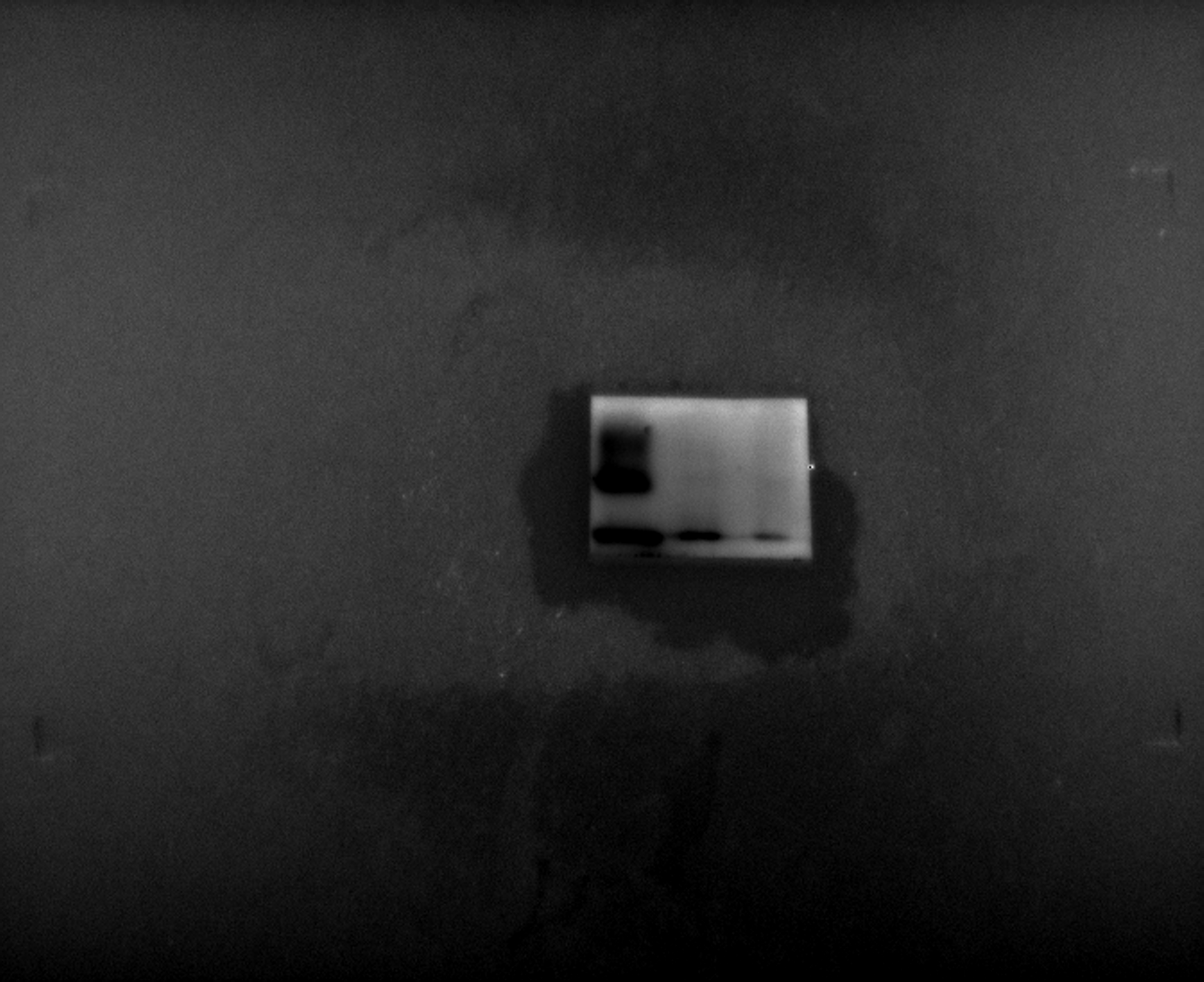

Supplement: Supplemental Material [file KBIE_A_2068754_SM7231.zip › supplementary/CO-IP/TSPO (3).tif]

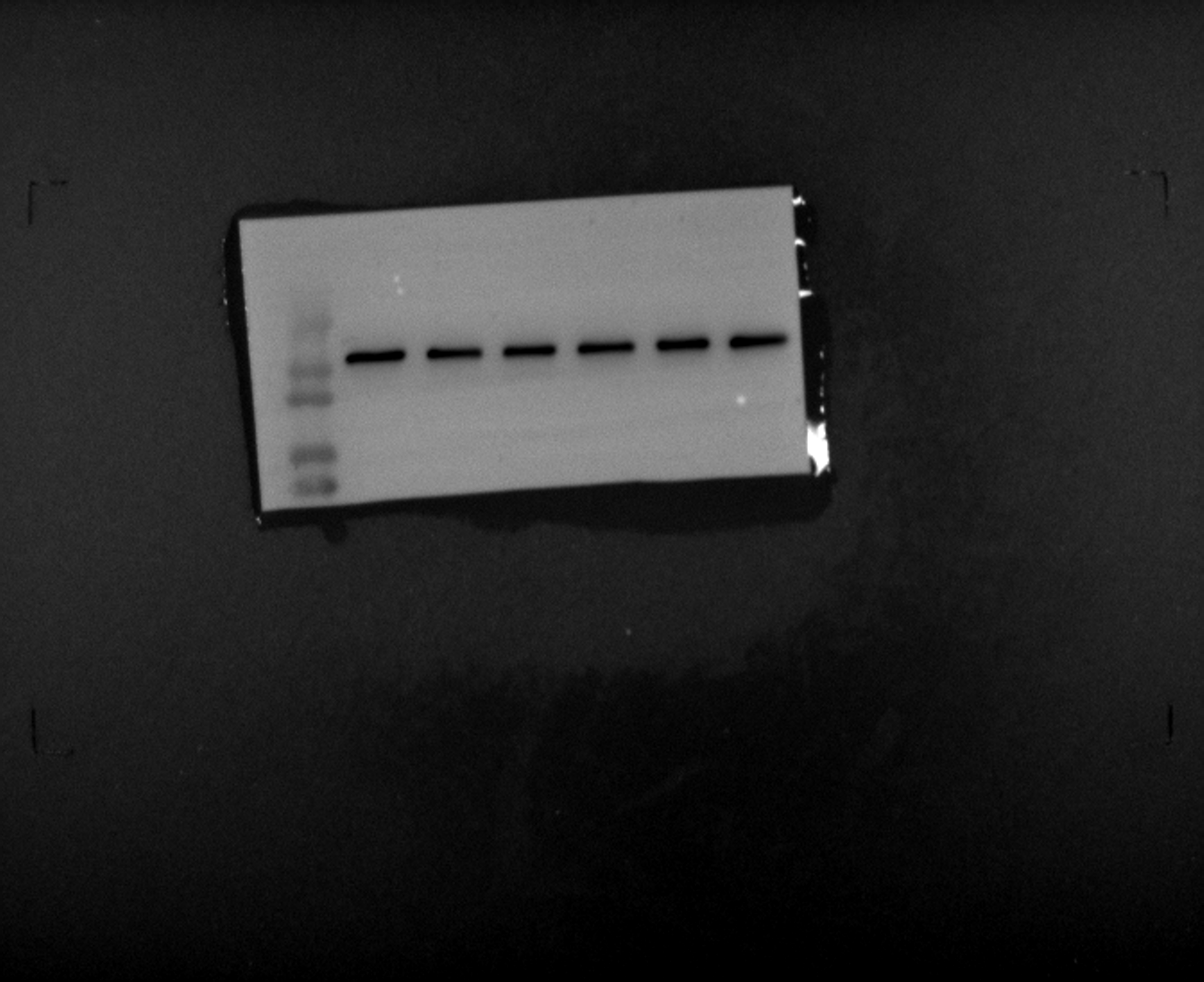

Supplement: Supplemental Material [file KBIE_A_2068754_SM7231.zip › supplementary/wb/GAPDH (1).tif]

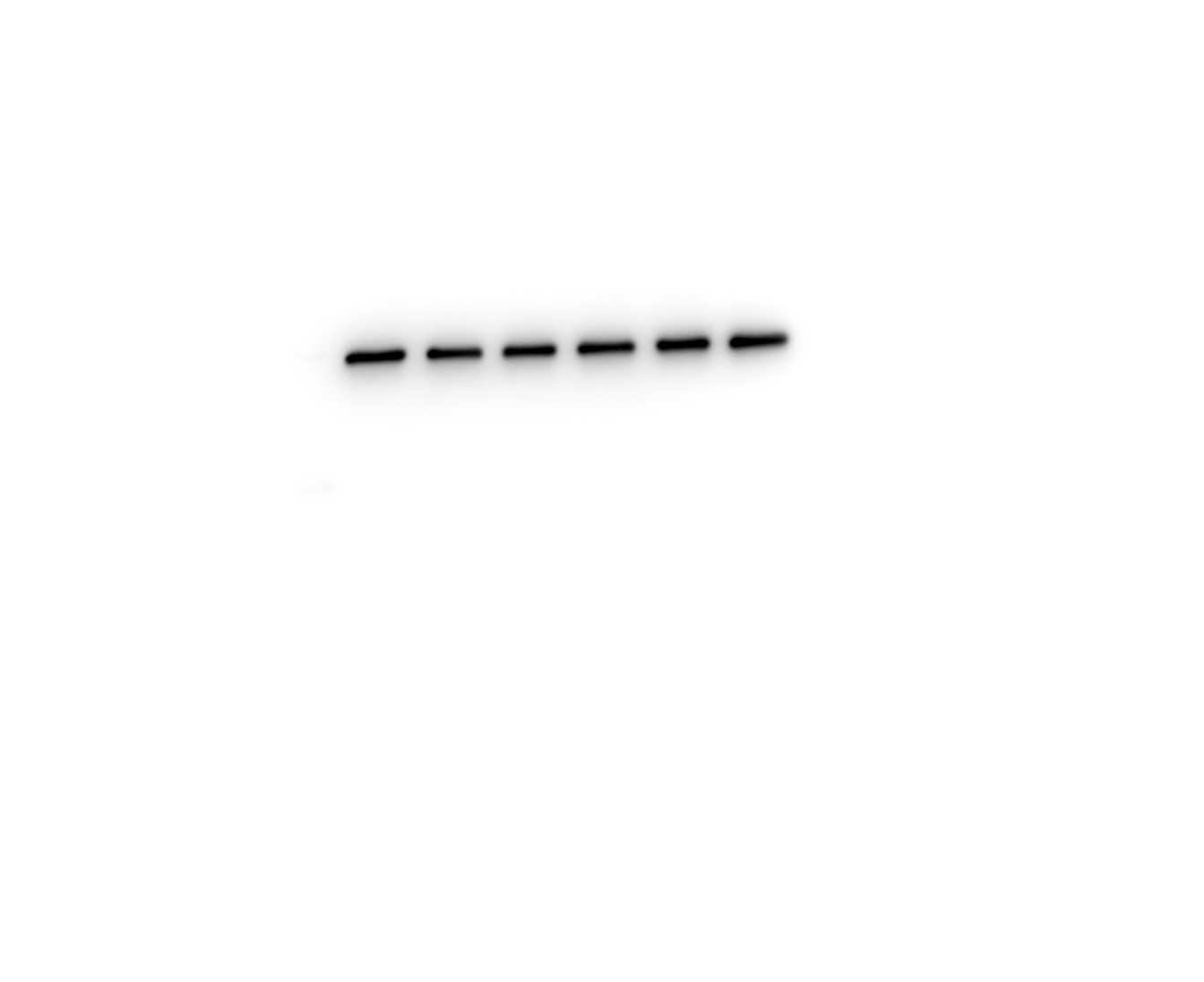

Supplement: Supplemental Material [file KBIE_A_2068754_SM7231.zip › supplementary/wb/GAPDH (3).tif]

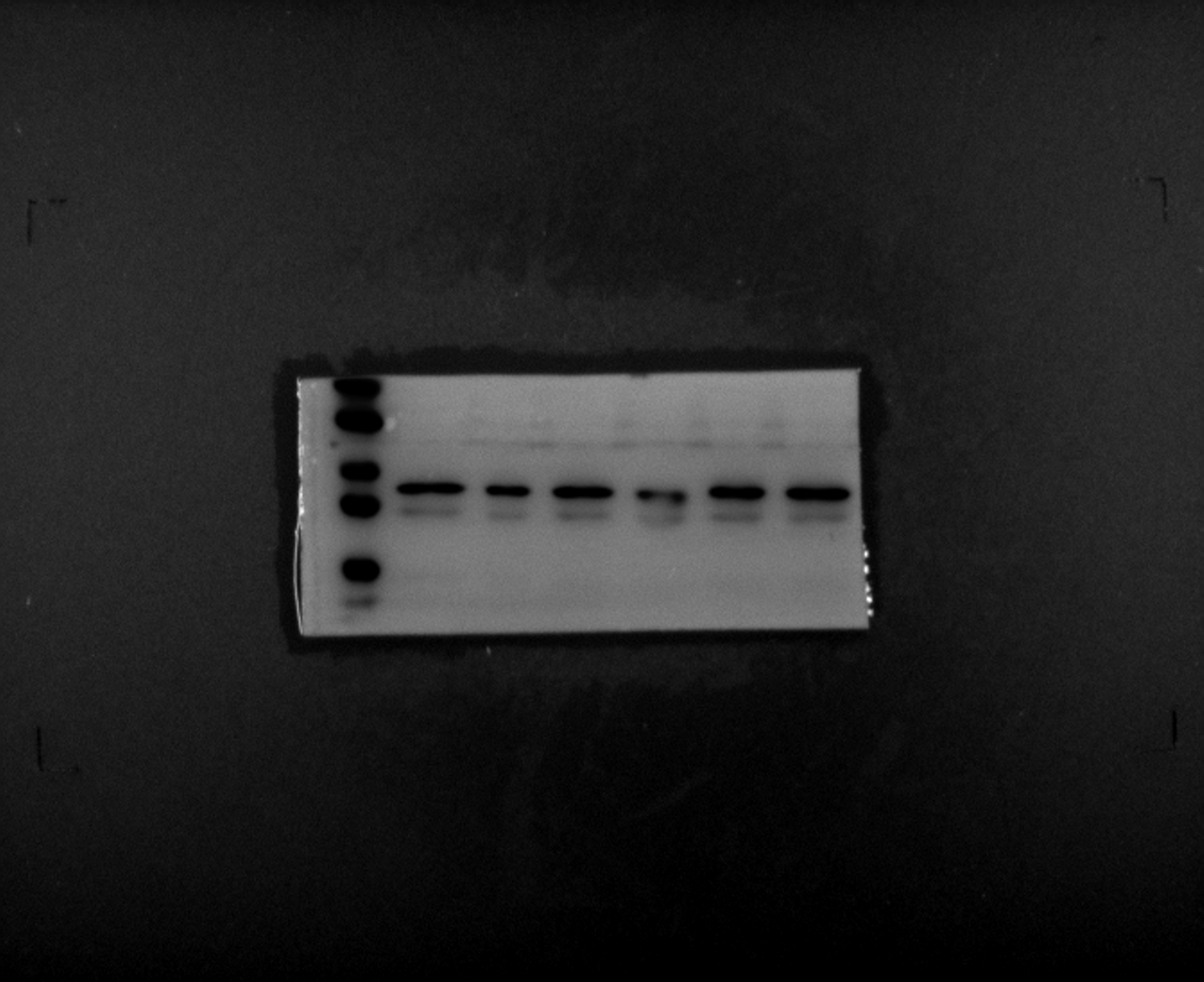

Supplement: Supplemental Material [file KBIE_A_2068754_SM7231.zip › supplementary/wb/bcl2 (4).tif]

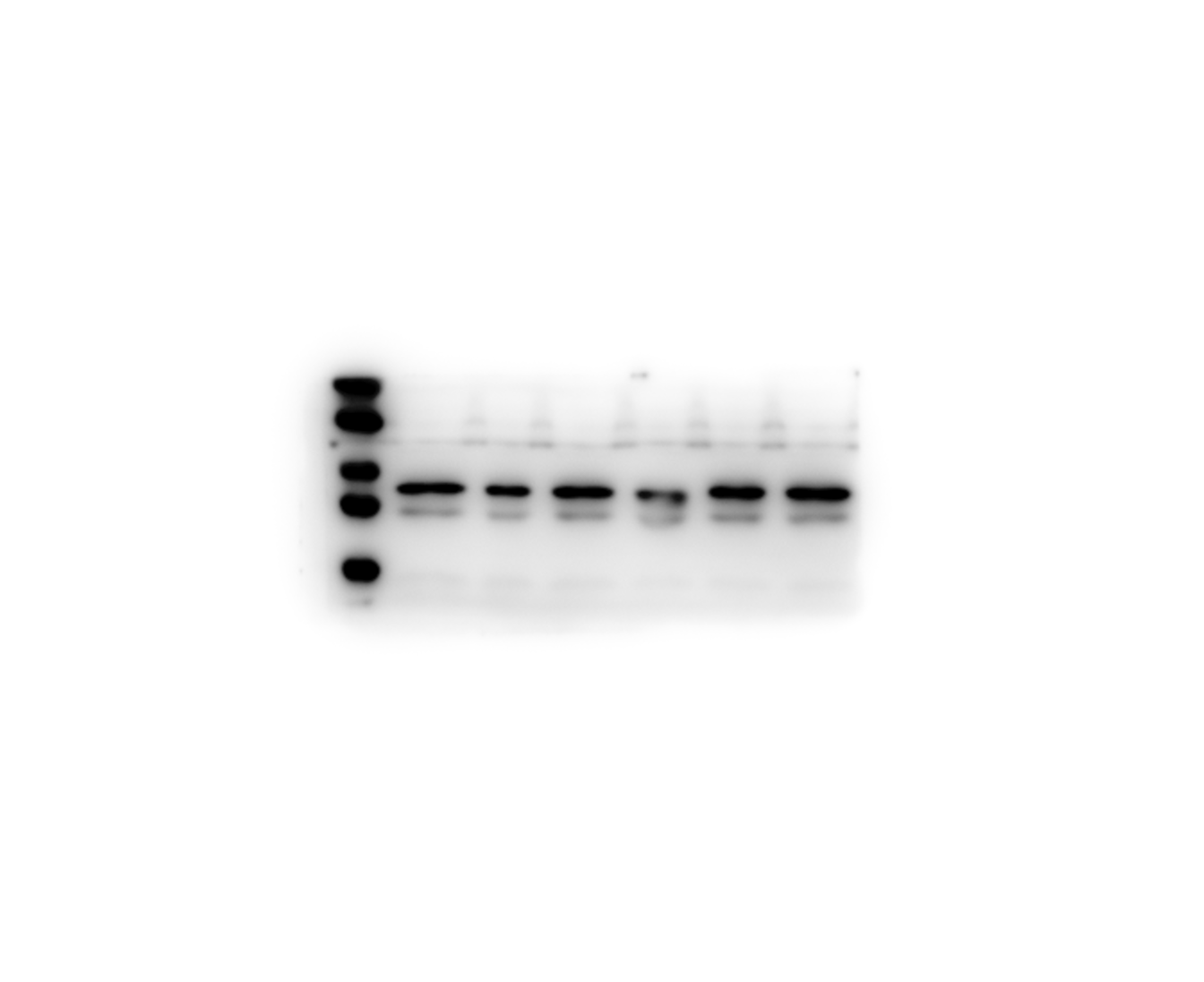

Supplement: Supplemental Material [file KBIE_A_2068754_SM7231.zip › supplementary/wb/bcl2 (6).tif]

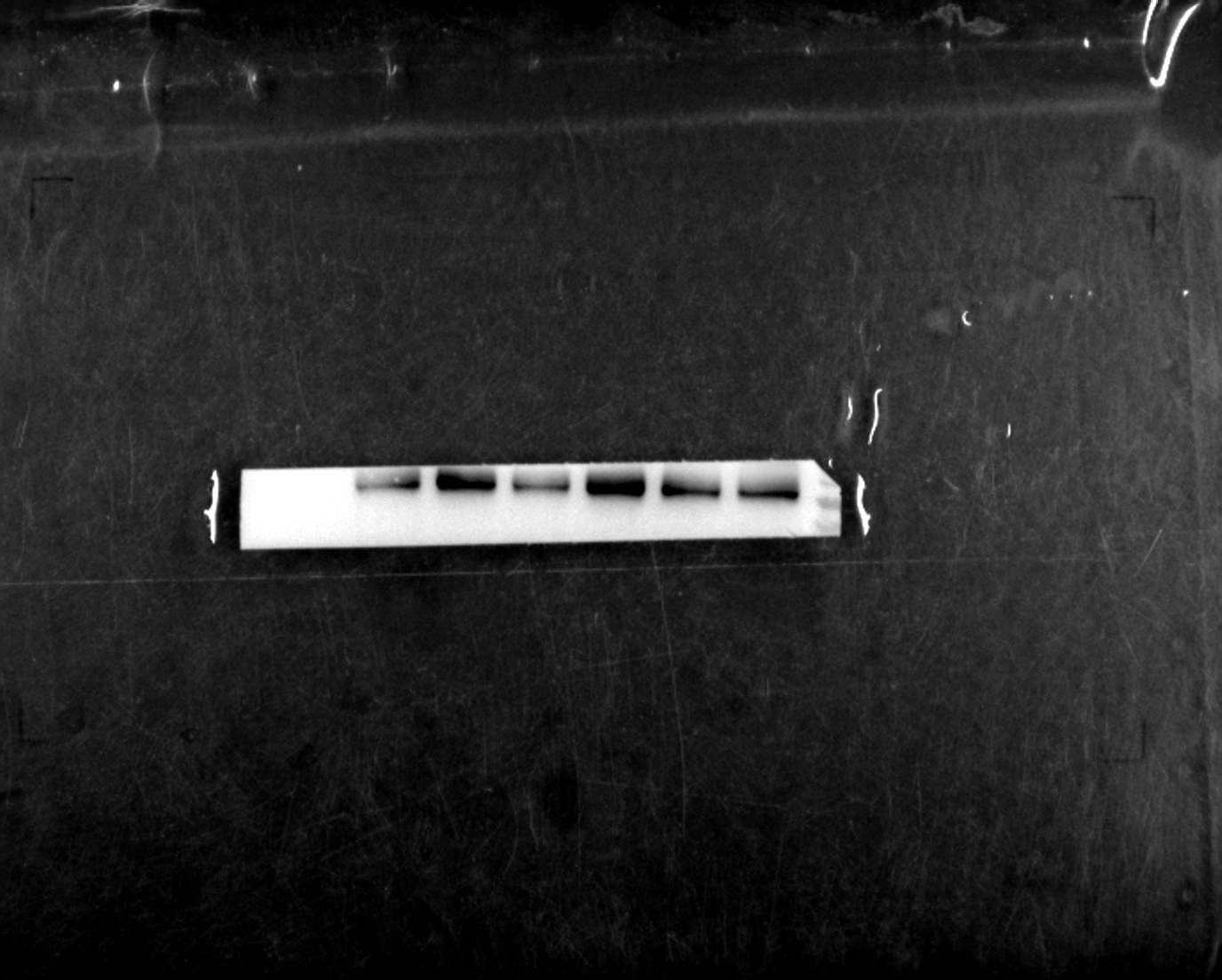

Supplement: Supplemental Material [file KBIE_A_2068754_SM7231.zip › supplementary/wb/caspase 3 (1).tif]

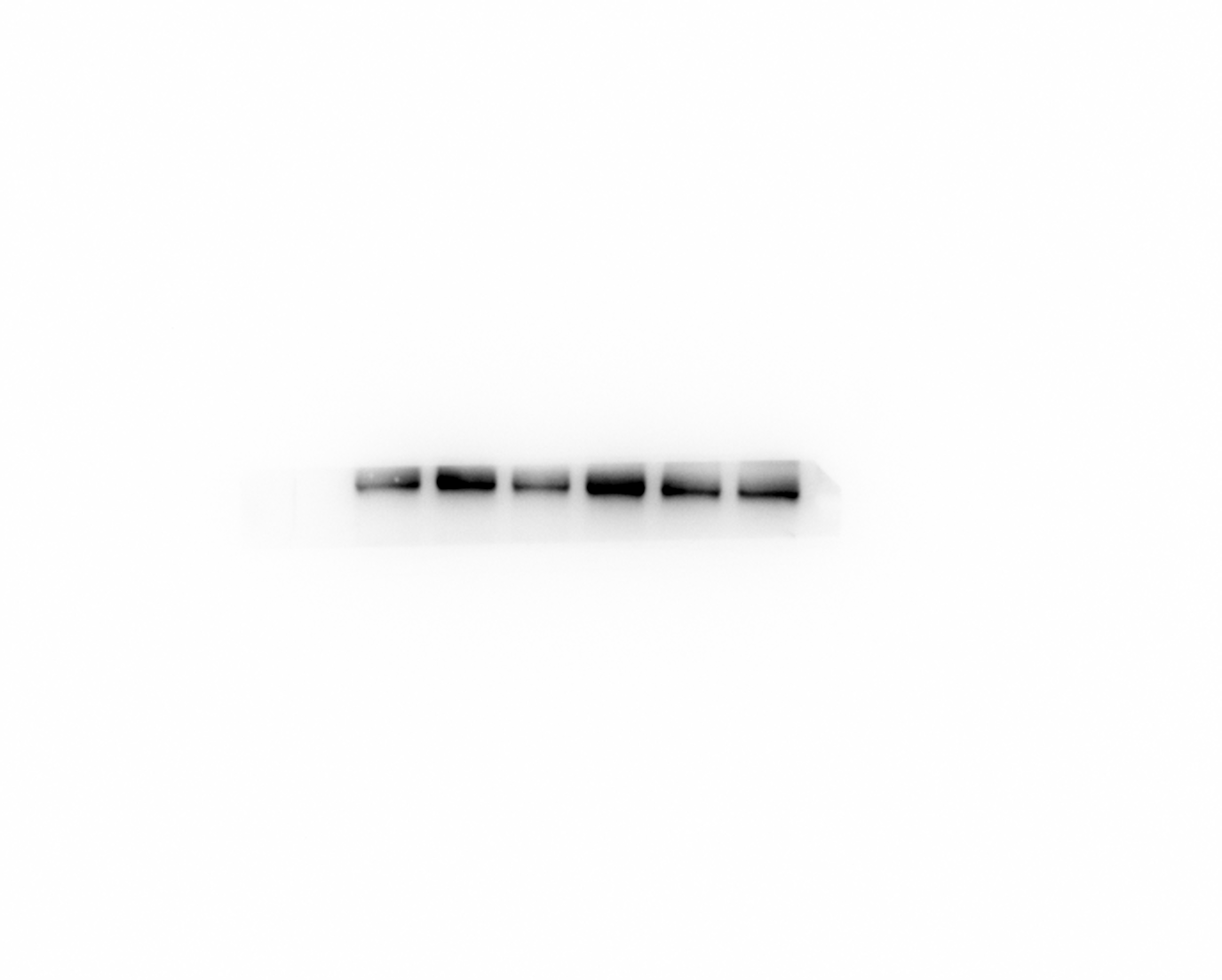

Supplement: Supplemental Material [file KBIE_A_2068754_SM7231.zip › supplementary/wb/caspase 3 (2).tif]
